# An exegesis of bacteriophage therapy: An emerging player in the fight against anti-microbial resistance

**DOI:** 10.3934/microbiol.2020014

**Published:** 2020-07-22

**Authors:** Oluwafolajimi Adesanya, Tolulope Oduselu, Oluwawapelumi Akin-Ajani, Olubusuyi M. Adewumi, Olusegun G. Ademowo

**Affiliations:** 1 Department of Medicine, College of Medicine, University of Ibadan, Ibadan, Nigeria; 2 Department of Medical Laboratory Science, College of Medicine, University of Ibadan, Ibadan, Nigeria; 3 Department of Virology, College of Medicine, University of Ibadan, Ibadan, Nigeria; 4 Department of Pharmacology & Therapeutics, College of Medicine, University of Ibadan, Ibadan, Nigeria

**Keywords:** bacteriophage, phage therapy, antimicrobial resistance, antibiotics, ESKAPE

## Abstract

Bacteriophages (simply referred to as Phages) are a class of viruses with the ability to infect and kill prokaryotic cells (bacteria), but are unable to infect mammalian cells. This unique ability to achieve specific infectiousness by bacteriophages has been harnessed in antibacterial treatments dating back almost a decade before the antibiotic era began. Bacteriophages were used as therapeutic agents in treatment of dysentery caused by *Shigella dysenteriae* as far back as 1919 and in the experimental treatment of a wide variety of other bacterial infections caused by *Vibrio* *cholerae*, *Staphylococcus* *sp.*, *Pseudomonas sp.* etc, with varying degrees of success. Phage therapy and its many prospects soon fell out of favour in western medicine after the Second World War, with the discovery of penicillin. The Soviet Union and other countries in Eastern Europe however mastered the craft of bacteriophage isolation, purification and cocktail preparation, with phage-based therapeutics becoming widely available over-the-counter. With the recent rise in cases of multi-drug resistant bacterial infections, the clamour for a return to phage therapy, as a potential solution to the anti-microbial resistance (AMR) crisis has grown louder. This review provides an extensive exposé on phage therapy, addressing its historical use, evidences of its safety and efficacy, its pros and cons when compared with antibiotics, cases of compassionate use for treating life-threatening antibiotic-resistant infections, the limitations to its acceptance and how these may be circumvented.

## 1. Introduction: The birth, rise and fall of the bacteriophage therapy concept

Abbreviations:

Bacteriophages are a group of viruses with the ability to infect bacteria without posing any threat to human host tissues or disturbing significantly the normal flora. *Phages*, as they are also known therefore have the potential to be used as anti-bacterial agents, theoretically speaking. The history of phage therapy can be traced to a time over a century back, and a full decade before the discovery of penicillin by Alexander Fleming in 1928 [1]. As far back 1896, a British bacteriologist Ernest Hanbury Hankin was able to prove that waters from Rivers Yamuna and Ganga contained biological compositions which possess the ability to pass through millipore filter meshes and destroy cholera causing bacterial cultures [2]. In 1915, Frederick Twort, a British microbiologist had described certain transparent organisms small enough to pass through filters with mesh sizes capable of trapping most bacteria [3]. It would be Félix d'Herelle, a French microbiologist who first described these organisms by the name: *‘bacteriophages’* and conceived the idea of using them therapeutically for the treatment of bacterial infections. This eventually led to the successful treatment of four pediatric cases of bacterial dysentery at the Hôpital des Enfants-Malades in Paris, producing the first documented example of phage therapy in 1919 [4]. For this feat, d'Herelle is widely regarded as the *‘father of phage therapy’.*

Despite his being the first to make the discovery, due to paucity of funds and his enlistment in the Royal Army, Frederick Twort was unable to further his research [3], a situation well capitalized upon by d'Herelle, who went on to carry out several non-randomized clinical trials of phage therapy all over the world, the results of which he captured in a 1931 report in the bulletin of the New York Academy of Medicine [5]. Félix d'Herelle eventually, through his collaboration with Georgian scientist George Eliava, established the Eliava Institute of Bacteriophage Microbiology and Virology (IBMV) in Tbilisi, Georgia in 1933, a facility that has developed into one of the most reputable bacteriophage therapy research centers in the world [6]. However, most of their works were written in Russian, and even after being translated into English, many of the results were rejected by the Western world as they were perceived to incompliant with international standards of safety [7]. While this did little to deter their work, d'Herelle and Eliava continued to isolate and characterize new bacteriophages, developing several therapeutic preparations which were sent to clinicians in different parts of the world, but their use was associated with very mixed results and low success rates. This fact, coupled with the boom in commercial development of antibiotics in the 1940s led to the widespread abandonment of phage therapy in the Western world [8], but not in Russia and Eastern Europe, due to the initial unavailability of antibiotics in the former Soviet Union [9],[10].

## 2. Phage biology and life cycle

**Figure 1.**
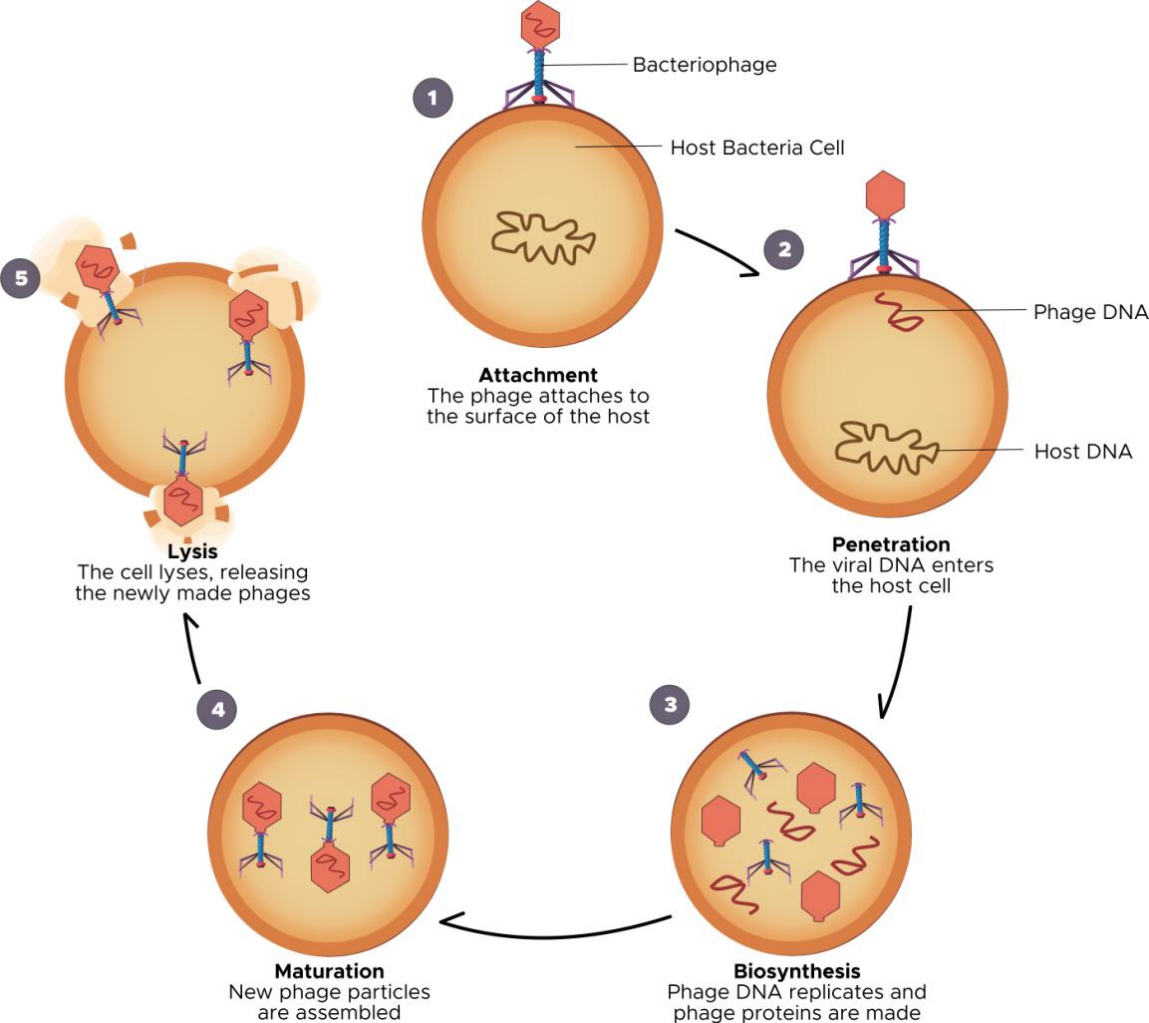
Bacteriophage lytic life cycle.

Bacteriophages are some of the simplest life forms, comprising of a genetic material, which could be DNA or RNA, enclosed within a protein coat (also known as: capsid). As simple as they may be structurally, bacteriophages are also incredibly diverse, representing some of the most ubiquitous living organisms in nature. It is estimated that there are between 10^31^–10^32^ bacteriophages in the world at any given time [11], helping to regulate population of bacteria in the natural ecosystem. Phages have also been said to be responsible for the elimination of 20%–40% of bacteria on marine surfaces every 24 hours [12]. To further contextualize their abundance, Keen [13] in a 2015 review retorted that there were approximately one trillion bacteriophages for every grain of sand in the world. Perhaps the only concept more ubiquitous than phages in life, is life itself! Phages have been isolated from numerous locations including: hospital surfaces, wastewater, human and animal feces and frankly anywhere else bacteria can be found [14],[15], and thousands of them have also been characterized, necessitating a robust system of classification, which employs criteria such as: morphological features, nucleic acid component, location and bacterial host [7]. A widely used method in literature classifies bacteriophages according to their biological cycles into either: lytic (or virulent) and lysogenic (or temperate) bacteriophages [16]. Bacteriophages are only infectious to bacteria because only they possess the receptors on their surface for phages to bind onto [17], and this goes on to determine phage host range. Bacteriophages bind to their host cell receptor (adsorption), and then they inject their genetic material into this cell. The difference between lytic and lysogenic phages is based on what happens next. Lytic phages are known to take over the bacterial replication machinery for the production of new copies (or progeny) of the phage within the bacteria. Multiple copies of these new phages are produced untill a *‘critical mass’* is reached, triggering the lysis of the bacteria cell wall and the release of the new phage progeny to reinitiate the lytic cycle (Figure 1) [16],[18],[19]. This critical mass (or burst size) usually depends on factors such as: specific phage characteristics, strain of bacteria infected by the phage and the environment within which the phage-bacteria interaction occurs [16]. On the other hand, lysogenic phages have been found to integrate their genetic material into that of the host, forming an entity called a *prophage*, allowing for the vertical transmission of genetic information of the virus to daughter cells of the bacteria following cell division, and possible expression of viral genes and proteins. Less commonly, the lysogenic phage genetic material does not integrate itself into the host bacteria's chromosome but remains intracellularly as a separate plasmid, which is still transmitted across bacterial generations. Under exceptional circumstances, environmental stress can induce a transition from a lysogenic cycle into a lytic cycle [20], though rare. Due to their ability to cause bacterial cell lysis, virulent phages possess the most therapeutic benefits, while lysogenic phages are strongly avoided due to certain capacities to propagate anti-microbial resistance [21], a concept soon to be explored in this review.

## 3. Perhaps the greatest public health emergency of our time

For many decades following their advent, pharmacological antibiotics revolutionized modern medicine, ending the possibility of global epidemics like the black death of the 14th century, which killed over 40% of Europe's population [22]; and an improvement in health indices, such as life expectancy, since the major causes of death used to be infectious diseases, mainly of bacterial origin [23]. Eventually, antibiotics became indispensable to modern medicine and although Fleming had predicted that without proper use, a time may come when bacteria develop sophisticated defense mechanisms against antibiotics, nobody expected that time to come so soon. Reports of resistance to penicillin began to emerge merely ten years after widespread use of the drug [24], and antimicrobial resistance (AMR) has since become one of the greatest public health emergencies of our time, according to the World Health Organization (WHO) [25] and the United Nations [26]. A 2013 review by Laxminarayan et al. [27] captures the devastating consequences of AMR brilliantly, with the United States Centre for Disease Control & Prevention (CDC) estimating that antibiotics-resistant infections result in approximately 2 million illnesses and 23,000 deaths per year, costing the United States (USA) $55 billion annually. In 2014, renowned British economist Lord Jim O'Neill, conducted a review on AMR. His landmark paper estimated that a total of 700,000 people die annually, globally from antibiotics resistant infections, and projects that number to hit about 10 million by 2050 [28].

The WHO in 2017, published the ‘*Priority Pathogens List’* [29], which is a list of twelve classes of bacteria, including *Mycobacterium tuberculosis*, which are adjudged to pose the greatest threat to human health due to their resistance to most of the existing treatment options. By ranking these organisms into: critical, high and medium priority, the goal of this publication was to serve as a guide for the discovery, research and development of new antibiotics for treatment of infections caused by these pathogens; however, due to the rapidity with which most antibiotics are now becoming redundant, there has been dwindling investment and interest, especially from the pharmaceutical industry in the discovery of new antibiotic drugs. Luepke et al. [30] reported that between 1983 and 1987, there were sixteen new antibiotics approved by the Food and Drug Administration (FDA). This number has drastically reduced, with only six new approvals occurring between 2010 and 2016, representing a 90% decline in antibiotics discovery. This is because of the exit of the big pharmaceutical companies from the field, due to regulatory and economic challenges that have made antibiotics development less lucrative.

Currently, there are about sixty new drugs in development, but even these bring little relief in the fight against AMR as very few actually target the critical-priority gram-negatives [31], including the notorious ‘ESKAPE’ pathogens (*Enterococcus faecium, Staphylococcus aureus, Klebsiella pneumonia, Acinetobacter baumannii, Pseudomonas aeruginosa* and *Enterobacter sp.*), responsible for the difficult-to-treat nosocomial infections. Furthermore, as most of these candidates are still in the pre-clinical phase of development, the regulatory hurdles they have to overcome before being available for widespread clinical use exclude them from the options available to us in the short term, as the ravaging consequences of anti-microbial resistant infections are already with us. There exists, abundant evidence that we have reached the end of the antibiotics pipeline, as the incidence of carbapenem-resistant, hospital-acquired *Klebsiella pneumonia* infections have risen significantly in the USA, and are associated with a 40%–50% mortality rate [32]. Although information about antibiotic resistance in Africa is very limited, it is expected to be more devastating due to high poverty index of the region, weak healthcare infrastructure and high incidence of antibiotic misuse and abuse [33]. All options need to remain on the table, if we are to survive the threat posed by AMR.

## 4. The resurgence of bacteriophage therapy, a familiar alternative

The early days of phage therapy were riddled with numerous mistakes, which made the results of clinical trials inconsistent and at times, non-reproducible. There was also a lack of scientific basis in historical data to serve as the working frame for phage preparation in western medicine [34]. These problems however could be attributed to widespread scientific and technological limitations of the time, including: poor understanding of phage biology and life cycle; underdeveloped purification and storage procedures, resulting in poor titers of active bacteriophages in therapeutic preparations and contamination of preparations with host bacterial antigens; and errors which therapeutic phage selection, resulting in the use of phages which were not effective against the target bacteria [3]. In addition, the many factors that determine the efficiency of delivery of phage preparations to the site of infection were not fully understood, for example, a report by Hodyra-Stefaniak et al. [35] recently established the effect of the immune system in causing phage clearance and concomitant reduction in the pharmacological concentration of administered phage therapeutics. However, with recent technological developments such as the invention of the electron microscope and advances in next-generation sequencing technology, we are able to better understand phage biology, and overcome the issues that had in times past blunted the prospects of phage therapy research. Bordet in 1925 described the phenomenon of bacteriophage lysogeny and lytic cycle induction [36] and this was also confirmed by Frank Macfarlane [37]. The invention of the electron microscope in 1931 enabled Helmut Ruska to describe the bacteriophage morphology between 1940 and 1943 [38]. This coupled with the urgent need for an alternative to antibiotics, has allowed for a resurgence of bacteriophage therapy research, and the prospects for development of phage-based anti-bacterial drug candidates. This is seen in the fact that as at 2019, the number of publications on phage biology available on PubMed increased to more than 600, compared to a total of 161 between 2007 and 2011 and approximately 15 at the turn of the century (1997–2001) [39]. Furthermore, there has been a surge in the number of clinical trials in human and animal models and cases of compassionate use, according to the Helsinki declaration, in life-threatening infections (summarized in Table 1, reviewed in [40]).

Human clinical trials for phage therapy date back almost one hundred years, with the earliest occurring in Eastern Europe, mainly the IBMV in Tbilisi, Georgia and the Ludwig Hirszfeld Institute of Immunology and Experimental Therapy in Wroclaw, Poland. At the Eliava Institute, various phage preparations were developed for the treatment of a wide array of bacterial infections, to a great deal of success. For example: ‘Pyophage’ was a preparation containing phages against *Staphylococcus aureus, Streptococcus proteus, Pseudomonas aeruginosa and Escherichia coli*, and was used to treat various skin infections, wounds as well as infections of the urinary and digestive tracts [41]. ‘Intestiphage’ is another preparation attributed with high success rates for the treatment and prophylaxis of gastrointestinal infections caused by: *Shigella sp., Salmonella sp., Proteus mirabilis, Pseudomonas aeruginosa* and enteropathogenic *E. coli* [41],[42]. During the 1974 typhoid outbreak in Rustavi, a cohort of 18,577 children (aged 4–14 years) was recruited for a prophylactic intervention trial using typhoid bacteriophage. A total of 8,732 children were administered the phage preparation systematically, 6,988 not systematically and 2,857 children were not given the treatment, which was a pill of bacteriophage (equivalent to 25 mL of liquid phage) per week. The results revealed a five-fold decrease in typhoid incidence among children who received systematic phage treatment, compared to the others. This trial results were well captured in an Eliava Institute report [43] of 1974.

One of the most comprehensive reports of phage therapy clinical trials was a compilation of results from the Phage Therapy Unit at Wroclaw, Poland, published in 2012 [44] by Miedzybrodski et al. Their trial, conducted between 2008 and 2010, involved a cohort of 157 patients, with drug-resistant infections ranging from: chronic bacterial prostatitis, deep tissue infections to osteomyelitis and otitis media. Phage preparations against: *Staphylococcus, Enterococcus, E. coli, Pseudomonas, Klebsiella, Enterobacter, Salmonella* and *Proteus*, were administered. Patient response to treatment was classified into seven categories, including ‘A’: pathogen eradication, ‘B’: good clinical outcome, ‘C’: clinical improvement, ‘D’: questionable clinical improvement, ‘E’: transient clinical improvement, ‘F’: no response and ‘G’: clinical deterioration. While categories A to C represented good response to treatment and constituted 39.9% (61 patients) of the results; categories D to G represented inadequate response to treatment and constituted 60.1% (92 patients) of the results. While these results do not re-inforce the case for phage therapy, they may be attributed to several factors as described by Nilsson [45] in a 2019 review. The trials were not randomized, double-blind clinical trials and involved many different types of infections, phage preparations and treatment procedures. Smith et al. also carried out a study on the use of bacteriophages in the treatment of acute diarrhea caused by *E. coli*, explained in [46]. Oral administration of bacteriophages targeting gut derived sepsis caused by *Pseudomonas aeruginosa* saved 66.7% of the mice from death [47]. Single strain of bacteriophage targeted against vancomycin resistant *Enterococcus faecium*, imipenem-resistant *Pseudomonas aeruginosa* and beta-lactamase producing *Escherichia coli* administered intraperitoneally was able to save 100% of mice in the bacteraemia models [48],[49]. In-vitro studies like that of Essoh et al. in 2013, have suggested the possibility of phage therapy as a treatment option for cystic fibrosis (CF). They employed bacteriophages purified from a Russian cocktail, targeting *Pseudomonas aeruginosa* in sputum samples of CF patients. These phages were tested against forty-seven sputum samples, of which lysis was evident in thirty-three samples [50]. Many more in-vitro studies around the use of phage therapy (with or without antibiotic combination) on cystic fibrosis patients suffering from acute to chronic lung infections caused by *Pseudomonas aeruginosa*, have been conducted, all yielding favourable results [41],[51].

**Table 1.** Showing a summary of the results from the published cases of compassionate phage therapy (cPT) *^X^Polymicrobial Infection; ^‡^Deceased; ENT: Ear Nose & Throat* [40].

Causative agent(s)	Site of infection	Route of administration	Number of patients	Concomitant antibiotics therapy	Outcome
*A. baumannii K. pneumoniae*	Bone	Intravenous	1	Yes	Success
*S. aereus P. aeruginosa E. coli Proteus*^x^	Bone; ENT; GI; Urogenital	Local; oral; rectal	15	Yes	Success (12/15 improved)
*S. aereus*	Bone	Intramuscular	1	Yes	Success
*S. aereus E. coli P. aeruginosa Proteus Streptococcus*	Urinary tract	Local	9	Yes	Success (decreased bacterial load in 6 patients and complete clearance in 3 patients)
*Achromabacter xylosoxidans*	Respiratory tract (Cystic Fibrosis)	Oral; inhalation	1	Yes	Success (Improved lung function)
*P. aeruginosa*	Respiratory tract (Recurrent Pneumonia)	Intravenous; inhalation	1	Yes	Success
*S. aereus*	Bone	Local	1	Yes	Success
*S. aereus P. aeruginosa^x^*	Bone	Local	3	Not reported	Not reported
*P. aeruginosa*	Bone	Local	1	Yes	Success in bacterial clearance^‡^
*E. coli Proteus S. aereus P. aeruginosa Streptococcus Enterococcus*	Skin (Burns, Ulcers)	Topical; sub-cutaneous	234	For some patients	Success
*P. aeruginosa*	Aortic valve graft	Directly through fistula	1	Yes	Success
*A. baumannii*	Brain (Post-operative cranial infection)	Intravenous	1	No	Success in bacterial clearance^‡^
*S. aereus*	Skin	Topical; oral	1	No	Intermediate success (decreased bacterial load, improved clinical state)
*A. baumannii*	Pancreas (Necrotizing pancreatitis)	Intravenous; local	1	Yes	Success
*P. aeruginosa*	Skin (Infected wound or septicaemia)	Intravenous; local	1	Yes	Negative blood culture but evidence of wound colonization remained^‡^
*P. aeruginosa*	Sepsis	Intravenous	1	Yes	Infection eradicated twice with subsequent regrowth^‡^
*S. aereus*	Skin (Diabetic toe ulcer)	Topical	6	Not recorded	Success (no amputation)
*S. aereus*	Eye (Corneal abscess)	Topical, inhalation, intravenous	1	Not recorded	Success
*P. aeruginosa S. aereus^x^*	Skin (Burn wound infection)	Topical	9	Prior to onset of therapy	Intermediate success in 8 patients
*Staphylococcus Enterococcus Pseudomonas E. coli Proteus*	Urogenital; Bone; respiratory tract	Topical; oral; vaginal; rectal; inhalation	157	Yes (29% of patients)	Success (18% of patients)
*P. aeruginosa*	Urinary tract	Local	1	Yes	Success
*Enterococcus faecalis*	Prostate	Rectal	3	No	Success
*S. aereus*	Gastrointestinal	Oral	1	No	Success
*P. aeruginosa*	Skin (Burn wound)	Topical	1	Yes	Success
*S. aereus*	Skin (wounds)	Topical	2	Yes	Success
*S. aereus E. coli P. aeruginosa Klebsiella^x^*	Sepsis	Oral	94	Yes (in 71 patients)	Success (85% of patients)
*Staphylococcus E. coli Proteus Streptococcus P. aeruginosa^x^*	Skin (Venous ulcers)	Topical	96	Yes	Success (70% of patients)
*S. aereus E. coli P. aeruginosa Klebsiella^x^*	Various infections	Oral; local	20	Not recorded	Success
*S. aereus E. coli P. aeruginosa Klebsiella Enterobacter^x^*	ENT; Urinary tract; respiratory tract; bone; skin (wounds); brain (meningitis)	Oral; topical; local	1307	Not recorded	Success (86% of patients)
					Intermediate success (11% of patients)

Phage bioengineering reveals opportunities for extended applications of phage therapy, through the deployment of CRISPR-Cas mechanism for the programmed disruption of antibiotic resistance genes and plasmids [52]. Furthermore, the isolation of phage lytic proteins: lysin and holins, which are very similar to eukaryotic lysozymes, allows for the development of ‘static’ phage-based pharmaceuticals. Both proteins are synthesized by bacteriophages, allowing the phage to lyse the bacteria cell wall for the release of viral progeny in lytic cycle.

**Figure 2.**
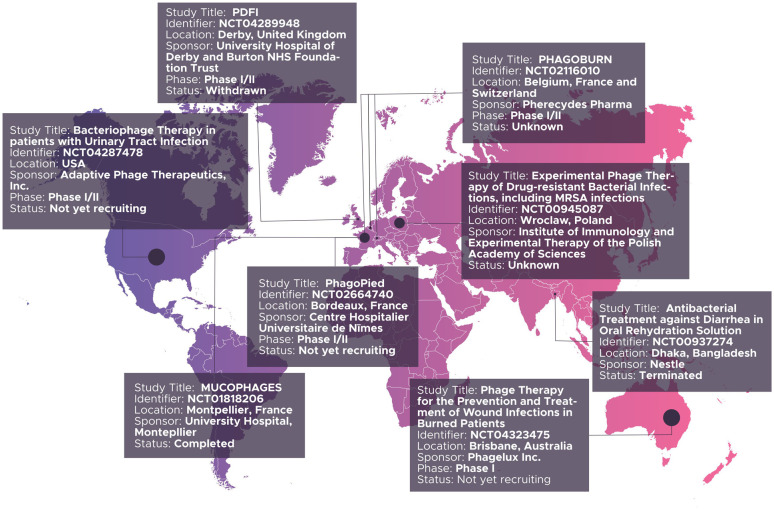
Summary of registered bacteriophage therapy trials as at April 2020.

In the west, several randomized, controlled clinical trials have been launched and described since 2000, especially in Europe. Two worthy of note include: a phase I trial to assess the safety of bacteriophage therapy for treating venous leg ulcers in humans. Rhoads et al. [53], in their 2009 paper reported no disparity in frequency of adverse effects between the test and control groups, thus establishing the safety of bacteriophage therapy. They however, recommended further phase II trials for efficacy in this case. Wright and colleagues, at the University College London conducted a Phase I/II randomized, double-blind, placebo controlled trial in 2009, which established the safety and efficacy of phage therapy for chronic otitis cases due to antibiotic resistant *Pseudomonas aeruginosa.* They administered a phage biopreparation, ‘Biophage-PA’, to a cohort consisting of a test group of twelve patients, for six weeks, and noted significant clinical improvement in the test group, as against the control group, also consisting of twelve patients [54]. Perhaps, the most extensive effort thus far is ‘Phagoburn’ [www.phagoburn.eu], described as the world's first multi-centric, randomized, double-blind, controlled clinical trial of phage therapy, ever performed according to both Good Manufacturing Practices and Good Clinical Practices [55]. Launched in 2013 and concluded in 2017, Phagoburn involved eleven partners in Belgium, France and Switzerland, with a focus on comparing the safety of efficacy of phage therapy (PP1131) against that of the standard of care (1% sulfadiazine silver emulsion cream) in burns patient with multi-drug resistant *P. aeruginosa* infections. In their report, published in 2018, Jault et al. [56], established that at low titres, phage therapy had a comparatively lower efficacy of treatment, compared to the standard of care. More recent and ongoing efforts include: ‘Phage4Cure’, launched in 2017, in Germany, with the aim of demonstrating the safety and efficacy of phage biopreparations in the treatment of antibiotic resistant *P. aeruginosa* infections in chronic Cystic Fibrosis (CF) or non-CF bronchiectasis patients [57]. Recently, AmpliPhi Biosciences Corporation, a biotechnology company based in Australia and the USA, launched a similar trial on *P. aeruginosa* infections in CF patients; intermediate results of which have established the activity of bacteriophages in removing biofilms of *P. aeruginosa* in chronic rhinosinusitis patients [58]. A search on https://clinicaltrials.gov using the other terms search phrase ‘phage therapy’ yielded fifteen results, of which nine were relevant to phage therapy. Figure 2, summarizes these results, including the status of each study.

Collectively reviewed, the results of the various clinical trials on phage therapy reflect an extensive variation in efficacy, making the push for bacteriophage therapy into mainstream and conventional clinical practice frustrating, especially in western countries. This however, calls for more studies, especially those comparing phage therapy with antibiotic therapy as well as exploring the efficacy of a combination of both therapeutic approaches. While phage therapy remains an integral part of the health system of many Eastern European countries, with many phage biopreparations being widely available for use in clinical medicine, not one phage-based medication approved for human use, has successfully made it to the Western European or USA market [59].

## 5. Bacteriophage therapy vs. Antibiotics: Pros and Cons

Bacteriophage therapy in theory and in practice is very different from antibiotic therapy, with major distinctions seen in their mechanism of action, pharmacokinetic and pharmacodynamic properties. These differences afford phage therapy some advantages over antibiotics as well as a number of limitations, which are systematically presented below:

### 5.1. On Specificity

Most bacteriophages are only able to infect bacteria hosts possessing complimentary receptors for phage antigen on their surface. This endows a special form of specificity to bacteriophages, and effectively determines the host range as established by Rakhuba et al. [17]. This host specificity also differs significantly across phage groups, with some exhibiting strain specificity and others possessing the ability to infect across several bacterial strains or even general [60],[61]. This specificity has been regarded as one of the most important advantages of phage therapy over antibiotics, because it affords phages a narrow spectrum of action, which prevents some problems attributed to antibiotics therapy, including: influence on normal flora with elimination of beneficial gut microbes, leading to development of secondary infections and emergence of resistant bacteria [62]. A very good example is antibiotic-associated diarrhea, usually secondary to resistant *C. difficile* infection, which have been attributed to the use of antibiotics such as: ampicillin, amoxicillin, cephalosporin and fluoroquinolones [63],[64]. Antibiotics use has also been implicated with the risk of development of asthma [65], obesity and diabetes [66],[67]. While the understanding of the effects of phage therapy on normal gut microbiome is limited at this time, several studies have reported the use of bacteriophages without modification of the microbiome in both animal models and in humans. In 2004, Chibani-Chennoufi et al. [68] reported the oral administration of four T4-like coliphages for the treatment of diarrhea-associated *E. coli*, to mice models, without modification of their gut microbiome. Similar confirmations have been obtained by Mai et al. [69], who used the phage preparation ‘ShigActive’, to treat *Shigella sonnei* in mouse models; and by Galtier et al. [70], who used a phage combination to treat uropathogenic *E. coli*, without disturbance of gut normal flora in murine models. In 2012, Sarker et al. [71], administered oral T4-phage preparations to human volunteers in Bangladesh, and reported no effect on liver, kidney and hematological functions.

While host specificity confers several advantages on phage therapy, it also possesses some limitations. As a result of this specificity, phage therapy could be less effective against polymicrobial infections, such as those found in infected burn wounds [71]. While this may be circumvented with the use of phage cocktails, containing multiple phages, the issue remains that for this strategy to work, clinicians need to have knowledge of which organisms are being treated, which is often not the case in empirical treatment strategies, highly favoring the use of broad-spectrum antibiotics. Furthermore, host specificity significantly limits the prospects of large-scale production and distribution of phage-based therapeutics. In a 2014 study, Bourdin et al. [72] tested T4-like phages obtained from two geographical locations (Mexico vs. Bangladesh), against *E. coli* strain isolates from each region. Their results reflected greater degree of coverage when the isolates were tested with phage samples obtained from the same region as opposed to when they were cross-inoculated with phage samples from a different region. The results from this in-vitro study were supported by findings from a randomized, placebo-controlled human trial by Sarker et al. [73] in 2016. In their own study, they administered orally, commercially-available Russian T4-coliphages or placebo, to a cohort of 120 Bangladeshi children with enteropathogenic *E. coli* infection, over four days. At the end of the trial, while the safety of phage therapy was established, it had failed to improve outcome in most of the participants, a result attributed to a failure of effective phage amplification at the site of infection. These two findings, both in-vitro and in-vivo, helped establish a concept that phage preparations may be best adapted to local microbial populations. Such regional specificity was further explained by the principle that phages targeting antibiotic-resistant bacteria strain would most commonly be found in the environment where the strains are abundant [74].

### 5.2. On safety

The adverse effects of antibiotics are well document in literature to include: neurotoxic, cardiotoxic, hepatotoxic, nephrotoxic as well as gastrointestinal and hematological effects. However, the most commonly reported are allergic reactions, attributed to drug interactions, intolerance or high-dose administration of the antibiotics [75]–[77]. Penicillin is one antibiotic which has been associated with severe anaphylactic reactions. Though extremely rare, when penicillin allergy occurs, it presents with a broad collection of symptoms including: nausea, pruritus, urticaria, wheezing, laryngeal edema and eventual collapse of the cardiovascular system, a phenomenon that has been extensively reviewed by Bhattacharya in [78]. In contrast, it is widely believed that bacteriophages are significantly safer and better tolerated, an opinion backed up by their specificity and inability to infect mammalian cells, as well as a notion that bacteriophages have existed alongside human life, and the likelihood of adverse reactions and allergies will therefore be low [4]. Evidence of bacteriophage therapy safety has been established by several clinical trials involving topical [41],[53],[54],[56] and oral [73],[79],[80] administration of phage cocktails, conducted in Eastern Europe, as well as the Western world. An important consideration for phage therapy is the ability of phages to migrate across the intestinal epithelium into the lymph, peripheral circulation and internal organs [81]. A study by Gorski et al., revealed that T4 phages are able to inhibit dendritic cell-mediated antigen processing and presentation function, which has been shown to extend the survival of skin grafts in mouse models [82]. This same study further suggested that phages are able to inhibit the production of other inflammatory cytokines such as: interleukin (IL)-2, tumor necrosis factor (TNF) and interferon (IFN) gamma, by mammalian white blood cells. These immunomodulatory properties potentially have the ability to maintain immune tolerance to foreign antigens derived from intestinal microbes, a principle that can be exploited for the treatment of inflammatory bowel diseases such as: crohn's disease and ulcerative colitis [83].

On the flip side, Tetz et al. [83], have shown that oral administration of a phage cocktail could increase intestinal wall permeability and levels of inflammatory cytokines in rat models. In their experiment, they administered commercially-available *Salmonella* bacteriophage cocktail as well as Pyobacteriophage, a Russian cocktail also commercially available, to five healthy albino Wistar rats, for ten days. They used the lactulose:mannitol ratio, as a marker for intestinal permeability [84], measuring its levels two days before the onset of the experiment and ten days after administering the phage cocktail. They found a significantly elevated lactulose:mannitol ratio, indicating an increased intestinal permeability. Their results reveal that phage therapy has the possibility of inducing intestinal barrier dysfunction and exacerbating the ‘leaky gut’ syndrome, a phenomenon that has been linked to a number of pathological conditions such as: crohn's disease and ulcerative colitis [83]. In addition, due to a scarcity of data on the use of bacteriophage therapy in immunocompromised patients, there have been concerns raised on their safety in this group of patients. However, Borysowski et al. [85], have reviewed the available literature, which suggests that phage therapy could also be a safe and efficacious treatment modality in immunocompromised patients.

### 5.3. On biofilm penetration

In biological systems, bacteria can be found either as planktonic (or free-living) organisms or as sessile organisms within complex ecosystems known as biofilms. Biofilms usually consist of bacteria cells, surrounded by an extracellular polymeric substance (EPS) matrix, and attached to an underlying surface (or interface) [86]. Wherever they are found, biofilms are associated with unique properties such as: reduced growth rates, the upregulation (or downregulation) of specific genes and quorum sensing [87]; making their eradication by conventional methods nigh impossible. While antibiotics are effective in the eradication of susceptible planktonic bacteria, they are extremely limited in the treatment of biofilm bacteria infections. Bacteriophages however, have been proven to be highly effective for the eradication of biofilms, as reported by Donlan [88] and Motlagh et al. [89]. This effect is achieved via an array of mechanism ranging from the infection and lysis of biofilm-forming bacteria, prior to attachment to an interface [90],[91], to the degradation of established biofilms through the production of an EPS depolymerase enzyme [92],[93]. This exposes the underlying bacterial population to lysis by the bacteriophages. An alternative mechanism involves the passage of bacteriophages through the water channels and pores which make up the heterogeneous structure; that is the biofilm extracellular matrix [94]. The efficacy of bacteriophage therapy against biofilms of *P. aeruginosa* was reported in a study by Gabisoniya et al. [95]; and against *E. coli* biofilm, even in a glucose-limited environment, was established by Corbin et al. [96]. Conversely, in order to penetrate biofilms, it would be required that most antibiotics be administered at high doses and for prolonged periods [97],[98]; and even this rarely eradicates the biofilms, and regrowth is common as soon as antibiotics therapy is ceased. As biofilms age, even higher doses of antibiotics would be needed for treatment, unlike in phage therapy, where biofilm age does little to alter the efficacy of treatment [99]. These findings make a strong case for the use of phage therapy, in the treatment of persistent infections caused by biofilm formation on implanted medical devices like: catheters, valves, prosthetics and lenses [88].

### 5.4. On development of resistance

AMR is a consequence of horizontal transfer of anti-microbial resistant genes (ARG) between bacteria, due to excessive use of antibiotic agents in human medicine as well as animal agriculture. A further compounding effect is the fact that the same ARG-possessing bacteria found in animals are responsible for the major zoonotic infections affecting humans, and they belong to the ‘ESKAPE’ group. This interaction has recently inspired the ‘One-Health Initiative’ movement, which considers the triad of human, animal and environmental health crucial, in the fight against AMR [100],[101]. Bacterial resistance to bacteriophages though unlikely, remains a possibility; however, it occurs through completely different mechanisms from bacterial resistance to antibiotics. Some of these include: mutation or loss of surface-receptors for bacteriophage binding; secretion of chemicals that prevent phage adhesion; blocking the injection of phage DNA into bacteria cell and inhibition of the phage replication and/or release processes as well as the integration of phage DNA into the clustered regularly interspaced palindromic repeats/CRISPR associated system (CRISPR/Cas) [102]. As far back as 1986, Riede et al. [103] demonstrated the interaction between TraT (an outer membrane lipoprotein) and OmpA (a membrane receptor which mediates phage binding), causing an inhibition of phage binding in *E. coli* strains. Similar mechanisms of surface receptor mutation have been observed in *S. aureus*, by Nordstrom et al. [104], in *Bordetella bronchiseptica*, by Liu et al. [105], and in *Vibrio cholera*, by Seed et al. [106].

Furthermore, Drulis-Kawa et al. [107], in 2012, documented the secretion of an extracellular polymeric substance (EPS) by environmental *Pseudomonas sp.*, and production of rods (alginates) and glycoconjugates by *Enterobacteriaceae;* both mechanisms preventing phage adhesion to bacterial targets. As a means of circumventing this possibility, therapeutic applications of bacteriophages usually involve the combination of multiples bacteriophages with low rates of bacterial resistance, into ‘cocktails’. This on its own presents a different set of considerations, as several factors need to be considered when developing phage cocktails for therapeutic use. These factors, reviewed by Chan et al. [108], include a need for prior knowledge of target bacteria, requiring standard microbiology laboratory investigations, which may not be an option due to limitations of time, cost or availability; and the dilemma of approach of either standardized (or ‘one-size-fits-all’) or customized phage cocktails. While standardized cocktails would be convenient, due to regional specificity of phage action (already described), their outcomes may differ across geographical regions; while the resources needed for production of customized phage cocktails are considerable, and have been regarded by some to be unsustainable. Rohde et al. [6], argue that bacteria resistance to bacteriophages is an unsustainable concept, for several reasons. First being the fact that the mechanism of resistance of microbes to bacteriophages, involves evolutionary mutations, which can be mimicked by phages to counter the development of resistance. While bacteria could alter their surface-receptors, phages could also evolve to recognize these new receptors, and also alter their genes, to prevent integration into bacterial CRISPR/Cas systems [109],[110]. A further advantage of the mutatory mechanisms of resistance to phages by bacteria was elucidated by Chan et al. [111], who reported that due to efforts to develop resistance to a lytic bacteriophage OMKO1, which targets surface receptor OprM for binding, mutations in OprM (a component of the multidrug efflux system; an antibiotic resistance mechanism), restored antibiotic sensitivity in multi-drug resistant *Pseudomonas aeruginosa* strains, in an evolutionary trade-off. This discovery presents exciting prospects for combination therapy involving antibiotics and bacteriophage cocktails. Second is a fact that even following development of resistance to a particular phage cocktail, new phages can simply be isolated from the environment of the bacteria to modify the contents of the cocktail, and restore sensitivity.

A different dimension in the discussion on resistance development is the possibility that phage therapy could indeed contribute to the development of resistance to antibiotics, in bacteria. Lysogenic bacteriophages incorporate their DNA into their hosts. As a result, theoretically speaking, they can help in the horizontal transfer of genetic material, especially the ARGs implicated in antimicrobial resistance, between bacteria [112]; and rightly so, as evidence of this abound in literature. In 2014, Marti et al. [113], were able to quantify six different ARGs–*bla_CTX-M_*, *bla_SHV_*, *bla_TEM_*, *qnrA*, *qnrB* and *qnrS*–using quantitative PCR, in both phage and bacterial DNA fractions of environmental water samples. A similar discovery was reported in 2015, by Rodriguez-Mozaz et al. [114], who found copies of ARGs like: *bla_TEM_* (conferring resistance against ß-lactams), *qnrS* (conferring resistance against fluoroquinolones), *ermB* (conferring resistance against macrolides), *sull* (conferring resistance against sulphonamides) and *tetW* (conferring resistance against tetracyclines), in hospital effluents and waste water treatment plant samples. Furthermore, Subirats et al. [115], reported similar findings, however, they also noted that the relative abundance of the ARGs in phage DNA fractions was higher than in bacterial DNA fractions (0.26% vs. 0.18%), pointing to the role of phages in acting as a reservoir for ARGs in nature. ARGs have also been found in bacteriophage DNA fractions obtained from human fecal sources [116]. The mechanism through which this process occurs is known as transduction, a phenomenon whereby phages mobilize bacterial genes, incorporating them into their own genetic material, and transferring these genes to another bacteria when they reinitiate their lytic cycle. Transduction is one of the many modes of horizontal gene transfer by bacteria alongside: conjugation, transformation and infectious transfer [117]. Clinically, a large number of ARG-carrying phages have been isolated in tissue samples and secretions of patients with recurrent infections due to antibiotic resistant microbes, which have previously been treated with antibiotics. In a study by Francello et al. [118], they read a total of 1,031 short sequences in the cystic fibrosis virome, from samples obtained from patients with antibiotic-resistant infections, and identified sixty-six efflux pumps, fifteen fluoroquinolone resistant genes and nine ß-lactamase genes. This evidence suggests that bacteriophages potentially act as vehicles for the perpetuation of multi-drug resistance in these patients.

The case against the use of lysogenic phages for therapeutic purposes is very strong, due to the evidences elucidated prior; however, with some genetic modifications, they have also been used successfully by some experts. Edgar et al. [119], prove that lysogenic phages could be used as vehicles for antibiotic-sensitive genes, in a similar way to how they convey ARGs. In their experiment, they were able to restore antibiotic sensitivity to streptomycin and nalidixic acid, by making use of a lysogenic phage to convey antibiotic-sensitive genes *rpsL* (for streptomycin) and *gyrA* (for nalidixic acid) into resistant strains of *E. coli.*

### 5.5. On administration

As a result of their unique mechanism of action, which involves the lysis of host targets, with the production of more phage copies, bacteriophages when being used for therapeutic purposed, do not require repeated dosing or administration to maintain their pharmacological therapeutic concentration, as compared with antibiotics (which require repeated administration over several days [120]. It is however important to mention at this point, that the unique pharmacodynamic and pharmacokinetic properties of phages, presents an important limitation to phage therapy; a phenomenon that has been well discussed in [45].

### 5.6. On cost

Evidence presented by Miedzybrodski et al. [121], suggests that indeed, phage therapy may provide more economic benefits to patients with antibiotic-resistant infections as compared with antibiotic therapy, as long as it isn't considered as a treatment of last resort. In their study involving a small cohort of six patients, including: two cases of methicilin-resistant *Staphylococcus aereus* (MRSA) causing osteomyelitis and pharyngitis; one case of methicilin-resistant coagulase-negative *Staphylococcus* (MRCNS) infection and three cases of methicilin-sensitive *S. aereus;* receiving oral phage preparation for a total of 6.5 weeks, the total cost of treatment was about 524 EUR. Comparing this with the total cost of a ten-day course of vancomycin (344 EUR–519 EUR), linezolid (1,559 EUR–1,890 EUR) and teicoplanin (2,620.85 EUR), they concluded that effective phage therapy for these infections in their Polish centre was cheaper than antibiotic therapy. Summaries of the pros (Table 2) of phage therapy are displayed.

**Table 2.** Potential benefits of bacteriophage therapy against antibiotic therapy

Criteria	Bacteriophage therapy	Antibiotics therapy
Specificity of action	Highly specific range of action	Broad spectrum of action
Toxicity	Almost completely non-toxic	Varying degree of toxicity ranging from mild to severe
Biofilm penetration	Effective penetrating ability	Unable to penetrate except in high doses
Possibility of resistance	Almost non-existent	High possibility of resistance
Ease of administration	Do not require repeated doses	Require repeated doses
Treatment cost	Potentially cheaper	Potentially more expensive

## 6. Phage therapy in Africa: current state of knowledge

Antibiotics misuse and abuse is rampant in many African countries, particularly those in sub-Saharan Africa. This is due to widespread poverty, lack of access to basic healthcare facilities in many rural settlements and inadequate knowledge among the populace of the dangers of antimicrobial misuse. These several socio-economic issues coupled with the shortage of adequate microbiological laboratory facilities for antimicrobial sensitivity and susceptibility testing, making empirical antibiotics use the mainstay of treatment even in healthcare settings, makes the burden of antimicrobial resistance on the continent to be very significant, with as much as 45% of mortalities in Africa, being attributed to infections by multi-drug resistant bacteria [122]. There is however a dearth of data on just how big of a problem AMR is in Africa, with a report by Tadesse et al. [123], showing that AMR data is unavailable in literature for 42.6% of African countries. In the light of these facts, bacteriophage therapy presents a potential game-changing situation in the fight against AMR infections on the continent, as bacteriophages can be used to develop novel antimicrobial agents that would not only be effective against these pathogens, but also be more cost effective and affordable for the many low-income healthcare settings found on the continent. However, following extensive literature search, only a few articles about bacteriophage therapy exploration in Africa were found, and almost all were focused on the identification and characterization indigenous bacteriophage types. In one report by Koko et al. [124], they described the discovery and characterization of fifteen morphological bacteriophage types, isolated from sewage, human fecal and surface water samples in Nigeria. These represent the first set of indigenous bacteriophages to be isolated from tropical Africa. Another study from South Africa [125] reported the characterization of lytic bacteriophages active against multi-drug resistant *Escherichia coli* 0177 strains isolated from cattle fecal matter. While a study reported the effective use of phage therapy against multi-drug resistant *Staphylococcus aureus* pneumonia in mice models [126], no study reporting the successful use of bacteriophage therapy on humans in a clinical setting in Africa was found. Some of the limitations against the exploration of phage therapy in Africa include: lack of financial resources as well as political will from the relevant government agencies to fund and approve such novel experimental endeavors. Furthermore, as is the case even on the global scene, existing regulatory frameworks guiding the development of medicinal products for use in human subjects, do not accommodate for the exploration of the bacteriophage therapy concept on the continent.

## 7. Advanced molecular applications of bacteriophage therapy

### 7.1. Use of polyvalent phages in multi-species biofilms

The use of bacteriophages for biofilm degradation is associated with a significant limitation. Due to the inherent mechanism through which biofilm-degradation by phages occurs, and the high host specificity of bacteriophages, a phage can ultimately only eliminate its target bacterial species from a particular biofilm community. This presents a unique consideration because most biofilms are composed of multiple bacterial species interacting at the molecular level to build the complex microbial ecosystem that often characterizes many biofilms. While the most obvious solution would be the use of a cocktail of bacteriophages, an alternative is the use of polyvalent phages (phages with broad or multiple host-ranges). Kim et al. [127] showed in 2012, that the polyvalent hage PA1Ø was effective in lysing a mixed-biofilm of *P. aeruginosa, S. aureus, S. epidermidis* and *S. hominis*. This capacity of polyvalent phages to lyse multi-species biofilms presents a unique opportunity for their expanded use in biofilm degradation, and more studies reflecting their application are abundant in literature [128],[129].

### 7.2. Application of iron-coupled phages and magnetic fields to penetrate biofilms

Adherence of microbial biofilms is mediated by chemical agents such as: adhesions and extracellular matrices, all of which possess an electric charge. It has long been hypothesized that application of a magnetic field strong enough to disrupt these essential kmolecules could in fact leave biofilms susceptible to antimicrobial agents. To test this hypothesis, Junka et al. [130] investigated the ability of antimicrobial agents such as: gentamicin, ciprofloxacin and chlorhexidine to penetrate and degrade biofilms of *S. aereus* and *P. aeruginosa* in the presence of a 10–50 Hz rotating magnetic field (RMP). They found that these antibiotic agents significantly penetrated the biofilms and inhibited the growth of the tested bacterial strains. They eventually concluded that a RMF could serve as an effective adjunct to antibiotic therapy for biofilm degradation. It could be hypothesized that this association would also hold true for bacteriophages and this was confirmed by Li et al. [131] in 2017. In their experiment, they coupled the polyvalent phage PEL1 to iron-based (Fe_3_O_4_) magnetic colloidal nanoparticle cluster (CNCs), which they then applied to newly-formed biofilms of *P. aeruginosa* and *Escherichia coli* C3000. They found that the iron-coupled, magnetized phage degraded 88.7% of the biofilm coverage area following 6 hours of treatment. This opens a new dimension to the application of bacteriophage therapy for the eradication of tough multi-drug resistant bacterial infections caused by biofilm accumulation.

### 7.3. Quorum sensing in bacteriophages

In 2019, Gallego del Sol et al. [132] described the molecular mechanisms behind phage communication systems, using the *Bacillus* phages as prototype. According to them, *Bacillus* phages make use of a communication system known as ‘arbitrium’, to communicate with themselves and their progeny, and coordinate the decision-making processes that determine whether a bacteriophage would carry out a lytic or lysogenic life cycle in its host bacterial cell. The arbitrium communication system is mediated by the production of a small peptide-AimP, six amino acids in length. This molecule promotes lysogeny, by inhibiting the negative regulator of lysogeny (AimX). Through this delicate system, bacteriophages are able to coordinate their own modes of expressions and switch between lysogenic and lytic roles.

### 7.4. Engineering bacteriophages: a magic bullet?

As advantageous as the phage therapy concept may be, the limited host specificity of many bacteriophages offers a significant limitation to their use in clinical settings where many infections are caused by a mixture of bacterial strains. While possible alternatives include the use of polyvalent phages or the use of phage cocktails or mixtures, phage bioengineering offers an additional option for consideration, as several techniques have been developed to engineer mutant phages with broadened or altered host ranges from wild-type phages. Yoichi et al. [133] successfully altered the host range of bacteriophage T2, by modifying the tail fibre genes (gp 37 and gp 38) of bacteriophage T2, and replacing it with the tail fibre genes of phage PP01 (which is specific for *E. coli*), through homologous recombination. A similar technique was used by Maichi et al. [134] to broaden the host range of bacteriophage T2. Another phage bioengineering technique uses the CRISPR-Cas systems for the modification of phage genomes. The type-1E-CRISPR-Cas system was used to engineer the T7 bacteriophage by Kiro et al. [135]. To achieve this, homologous recombination was used to delete the T7 gene 1.7 which was considered non-essential to the bacteriophage. A CRISPR counterselection system was then employed to enhance the growth of the recombinant phage genome, resulting in the cleavage of non-recombinant phage genome containing the non-essential gene 1.7 [134]. Phage bioengineering presents exciting prospects for bacteriophage therapy, as it can be used to proffer solutions to many of its limitations.

## 8. Regulatory hurdles facing phage therapy, efforts so far and the future of phage therapy.

Due to the lack of a global framework for the adoption of phage therapy into conventional medicine, there are several regulatory hurdles the concept has to overcome along its way to widespread acceptance. These challenges are fuelled by its position at the apex of personalized medicine, making it unconventional, breaking existing moulds of pharmaceutical regulatory policies. The issue of intellectual property rights on phage therapy medicinal products (PTMPs) need to be resolved, as bacteriophages obtained from nature cannot be subjected to patent rights, presenting a unique dilemma against the commercial production of phage cocktails. Further, the lines remain blurred concerning the need for market authorization for PTMPs, as some argue that they are formulated in the laboratory for specific patient (magistral) use, not involving an industrial process. On the other hand, large-scale production would involve industrial processes requiring market authorization as stipulated under Directive 2001/83/EC guiding the licensing of medicinal products for human use in Europe [136]. Finally, their unique pharmacodynamic properties as ‘active drugs’, with the potential to self-replicate in the body offers additional layers of consideration to be addressed going forward.

There are however signs of progress with the implementation of a pragmatic framework for PTMP magistral formulations in Belgium, which involves the use of a monograph to assess the standards of active substances to be used in PTMPs, followed by the testing of these substances against the monograph standards in an accredited laboratory, after which a pharmacist may proceed to formulate the PTMP in line with a physician's prescription [137]. This however shifts responsibility for the formulated compound to the pharmacist and prescribing physicians. Similar frameworks have been implemented in France: Authorization Temporaire d'Utilisation nominative (ATUn) [138] and the United States of America (USA): under the emergency investigational new drug (eIND) pathway [139]. Indeed, it would seem that under the immense pressure exerted by anti-microbial resistance, many are beginning to awaken to the potential benefits of bacteriophage therapy. The reality remains however that many of these so-called new regulations are reminiscent of those implemented in countries that never abandoned phage therapy for antibiotics in the first place. This suggests that we may have more to learn from them, not only in research and development, but also on policy and legal considerations, if phage-based therapeutics are to gain a foothold in modern medicine.

## 9. Conclusion

The evidence supporting the use of bacteriophages as either a therapeutic alternative or an adjunct to antibiotics is unquestionable. Numerous studies carried out in vivo and in vitro have confirmed the safety and efficacy of bacteriophages in the treatment of multi-drug resistant infections. However, more studies are required for a proper understanding of the unique pharmacological properties of phages, which affect considerably their use in clinical practice. Similarly, their immunomodulatory functions, potential for resistance and their role in perpetuating antibiotic resistance, require more elucidation. In spite of these, the future of phage therapy is bright. Their many advantages over antibiotics and frankly speaking, the burden of AMR ensures this fact at the very least.
